# Rates of Preeclampsia and Post-preeclamptic Cardiovascular Disease Among US Military Servicewomen: A Retrospective Case-cohort Study

**DOI:** 10.1093/milmed/usad300

**Published:** 2023-08-04

**Authors:** Thornton S Mu, Amelia Duran-Stanton, Emily A Stone, Lee Ann Zarzabal, Andrea Loewendorf

**Affiliations:** Brooke Army Medical Center, JBSA Ft. Sam Houston, TX 78219, USA; Army Medical Center of Excellence, JBSA-Fort Sam Houston, TX 78234, USA; Defense Health Agency, San Antonio, TX 78205, USA; Defense Health Agency, San Antonio, TX 78205, USA; ImmunoVation, Stanton, CA 90680, USA

## Abstract

**Introduction:**

Preeclampsia (PE), a hypertensive-inflammatory disorder of pregnancy, poses acute risks of seizures, stroke, and heart attack during pregnancy and up to 6 weeks post-delivery. Recent data suggest that residual increased risks for cardiovascular disease (CVD) linger for much longer, possibly decades, after PE pregnancies. In civilian studies, PE and the major vascular events resulting from it disproportionately affect women from minority groups, especially African American women. The Military Health System (MHS) provides equal access to care for all active-duty servicewomen (ADSW), thus theoretically mitigating disparities. Racial/ethnic breakdown for PE and post PE CVD has not been studied in the MHS.

**Materials and Methods:**

We identified healthy pregnancies in the MHS electronic health records of ADSW in the years 2009/2010 and those with a PE diagnosis. Patients with preexisting conditions of PE or CVD based on a look-back period of two calendar years were excluded. Cases were matched to controls based on age at pregnancy within 5 years and race/ethnicity. Cohort was assessed for diagnosed CVDs, race, age, and service during 2011–2017. Time to first CVD event was assessed with Cox proportional hazards model, results reported as relative risks (95% CI). All variables were summarized using mean (SD) for normally distributed continuous variables; non-normal continuous variables were characterized by median [IQR] and categorical variables were summarized by counts and frequencies. All statistical testings were two-sided with a significance level of 5% and were completed using SAS-EG version 9.2 or R version 3.5.2.

**Results:**

From an analysis of 106,808 inpatient ADSW records, PE incidence by race is 11.8% for White, 12% for African American, 11.4% for Asian/Pacific Islander, 11.2% for Native American, 9.5% for Other, and 7.6% for unknown (not documented) race. Thus, in the US Military, African American women have comparable (0.2% higher) PE rate than White women in contrast with civilian studies that often report much higher incidence in the African American population. Using Asians as referent group, PE increases the risk of CVD. White women have a hazard ratio (HR) of 1.47 95%CI (1.15–1.88), African Americans a HR of 1.51 95% CI (1.18–1.93), and Other a HR of 1.39 95% CI (1.01–1.91).

**Conclusion:**

In this study, we report overall higher incidence of PE in military women than what is published for civilian women in all races and across all services. Importantly, we do not find significantly higher numbers of PE and post-PE CVD for African American, compared to White women in the military. Our study is not designed to address differences between military and civilian PE epidemiology, but these results deserve further exploration. This study shines light on a health risk unique to women, which we found to be more prevalent in the US Military than published civilian population. Further study to determine the details of long-term morbidity, disability, and death attributable to PE (CVD, stroke, and kidney diseases) are needed to design optimal medical management protocols, ensure readiness for duty, and protect our Women Warfighters.

## INTRODUCTION

Preeclampsia (PE) is a hypertensive-inflammatory pregnancy disorder of unknown origin that can result in serious morbidity and mortality for both mother and fetus, and PE complications continue after birth. PE is associated with ∼4 fold higher incidence of chronic hypertension, heart disease, and stroke post-pregnancy and is more predictive of cardiovascular disease (CVD) than obesity.^1^ African Americans generally have a higher prevalence of coronary heart disease, cardiovascular death, and present with greater clinical severity independent of PE. PE rates in civilian populations are usually reported around 3–8% with higher rates for Black women (African and African American) than other races.^2,3^

CVD is the most common cause of death among women over 50, and stroke is the third leading cause of death in women worldwide.^4^ In 2018, O’Donnell et al. wrote “The identification of CVD risk factors, including others not studied in this analysis [preeclampsia was not included], offers the opportunity for preventative interventions that can reduce the rates of clinical CVD during, but especially after, military service.”^5^ Lastly, veteran status—*per se*—is a risk factor for CVD.^6^

Therefore, we examine a dataset of US active-duty servicewomen (ADSW) with PE and subsequent post-PE CVD prevalence in the 10 calendar years after delivery compared with ADSW with pregnancies without PE. We then discuss these observations in comparison with other US and international datasets.

## MATERIALS AND METHODS

### Ethics Statement

This study was approved by the Institutional Review Board (IRB) of the Brooke Army Medical Center Reference# C.2018.139 n.

### Source Data

We conducted a retrospective case-control study from the Military Health System (MHS) inpatient electronic health records of pregnant ADSW in the years 2009–2010, specifically collecting data on new episodes of PE (YES/NO) during that time period, with matched pregnant case controls based on age and race/ethnicity and service branch. PE diagnosis was defined with International Statistical Classification of Diseases 9 (ICD9) and ICD10 codes (Table I). We excluded outpatient records and patients who had preexisting conditions of PE or CVD conditions based on a look-back period of two calendar years. We identified a total number of 2,288 cases of inpatient records with PE using these criteria for case-control matching. Cases and controls were matched based on Service branch, age group (less than or equal to 24 years, 25–29, 30–34, and 35+), and race (White, African American, Asian/Pacific Islander, Other). After matching, we removed 46 patients who were enrolled from the Public Health service and 76 patients with unknown or missing race information.

**TABLE I.** ICD Codes Used for the Identification of Pregnancies, PE/E, and CVD/VD

Condition	ICD9	ICD10
Births	V270, V272, V273, V275, V276, 650	Z370, Z372, Z373, Z375, Z3751, Z3752, z3753, Z3754, Z3759, Z3760, Z3761, Z3762, Z3763, Z3764, Z3769
Abortion/Miscarriage	634x (spontaneous abortion/miscarriage) 635x (legally induced abortion) 636x (illegally induced abortion), 637x (unspecified abortion), 638x (failed attempted abortion), 639x (complications due to abortion)	O00x-O08x (ectopic pregnancy, hydatidiform mole, other abnormal products of conception, spontaneous abortion, complications after induced termination of pregnancy, failed attempt at abortion, complications due to ectopic & molar pregnancies)
Preeclampsia/eclampsia	6,424, 64,241, 64,242, 64,243, 64,244, 6,425, 64,251, 64,252, 64,253, 64,254, 6,426, 64,261, 64,262, 64,263, 64,264, 6,427, 64,271, 64,272, 64,273, 64,274, 6,429, 64,291, 64,292, 64,293, 64,294, 6,427, 78,039	O14, O140, O1400, O1402, O1403, O141, O1410, O1412, O1413, O142, O1420, O1422, O1423, O149, O1490, O1492, O1493, O15, O150, O1500, O1502, O150, O151, O152, O159
CVD (chronic hypertension; ischemic heart disease; cerebrovascular disease, thromboembolism, Cardiomyopathy; Ischemic Heart Disease; Myocardial Infarction; Coronary Heart Disease; HELLP syndrome)	93.9, 401, 401.1, 401.9, 402, 402.1, 410, 410.01, 410.02, 410.1, 410.11, 410.12, 410.2, 410.21, 410.22, 410.3, 410.31, 410.32, 410.4, 410.41, 410.42, 410.5, 410.51, 410.52, 410.6, 410.61, 410.62, 410.7, 410.71, 410.72, 410.8, 410.81, 410.82, 410.9, 410.91, 410.92, 411, 411.81, 412, 413, 414, 425, 425.1, 425.11, 425.18, 425.3, 425.4, 425.5, 425.7, 425.8, 430, 431, 432, 432.1, 432.2, 432.3, 432.4, 432.5, 432.6, 432.7, 432.8, 432.9, 434.01, 434.1, 434.11, 434.91, 436, 440.9, 452, 453, 453.3, 453.4, 453.41, 453.42, 453.5, 453.51, 453.52, 453.6, 453.72, 453.73, 453.74, 453.75, 453.76, 453.77, 453.79, 453.81, 453.82, 453.83, 453.84, 453.85, 453.86, 453.87, 453.89, 453.9, 557, 799.89, 997.02	I10-I30; I33-I40; I42-I50; I60-I72; I74; I80.1-I80.3

IHD, Ischemic Heart Disease; GH, gestational hypertension; MI, Myocardial Infarction; CV, cardiovascular; CVD, Cardiovascular Disease; HELLP, Hemolysis Elevated liver enzymes Low Platelet counts; PE, preeclampsia; E, eclampsia.

### Statistical Analysis

Time to first CVD event in the follow-up period after the first pregnancy/PE delivery was assessed with a Cox proportional hazards model. We conducted additional post-hoc analysis to assess if there was a significant difference between the PE cohorts as a function of race, age, or branch of service. The final matched study cohort was assessed for diagnosed CVDs and different service-level variables of interest during the follow-up period of Calendar Year 2011–2017 using the ICD codes listed in Table I. Results were reported as relative risks (95% CI) with comparison of risk between race/ethnicity groups. All variables were summarized using mean (SD) for continuous variables that were normally distributed. Nonparametric continuous variables were characterized by median [IQR]. Categorical variables were summarized by counts and frequencies. All statistical testings were two-sided with a significance level of 5% and were completed using SAS-EG version 9.2 or R version 3.5.2. Graphical depiction was accomplished with PRISM (Graphpad, La Jolla, CA) or Excel (Microsoft).

## RESULTS

### Records Used in This Study

Between January 1, 2006 and September 30, 2017, the combined number of records of pregnancies, miscarriages, and abortions was 126,454 for military care inpatient and 59,047 for civilian care inpatient. These data included ADSW from the Army, Navy (including Coast Guard and Marines), and Air Force.

**FIGURE 1.**
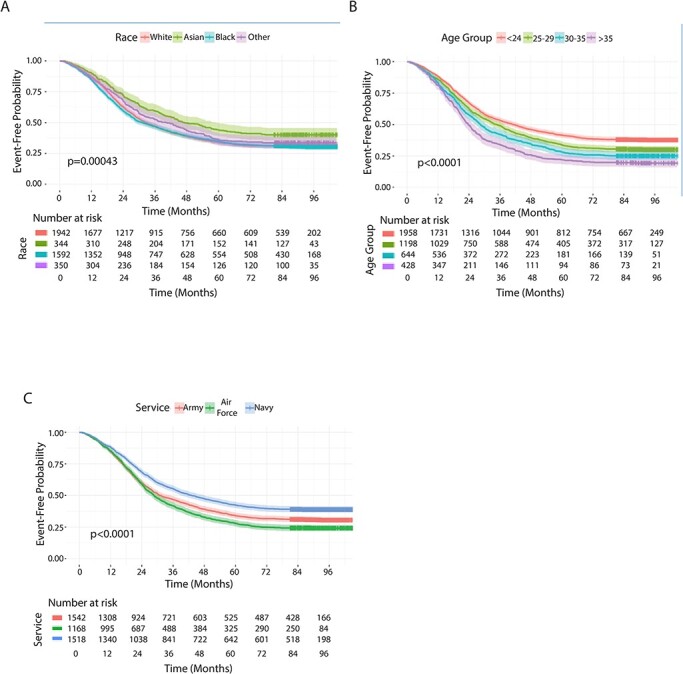
Cox proportional hazard model analysis of Military Service Women Post-PE risk of CVD event and confounding factor (A) race, (B) age, (C) service branch.

A total of 2,288 PE cases from the inpatient records were assessed for CVD or PE in the two calendar years prior to the index pregnancy and removed from the study accordingly (see materials and methods, Fig. 1). After exclusion of patients in the Public Health service or with unknown/missing race information, 2,166 women with PE met inclusion criteria and were matched by age (within 5 years) and race with healthy pregnancies/miscarriages.

### PE Prevalence

The overall prevalence of preeclamptic pregnancies from only inpatients was 12,364 out of 106,808 or 11.2% between 2009 and 2017. This group was used for further analysis of the racial breakdown of PE prevalence in the United States Armed Forces, which shows African Americans having the highest PE rate (12%) followed by Whites (11.8%), Asians/Pacific Islanders (11.4%), Native American Women (11.2%), other (9.5%), and unknown with 7.6% (Table II).

**TABLE II.** Preeclampsia Prevalence by Race from Distinct Inpatient Encounters, 2009–2017

Race	Preeclampsia	All pregnancies	Percentage
White	6,607	55,979	11.8
Asian/Pacific Islander	1,083	9,492	11.4
African American	3,555	29,593	12.0
Native	262	2,323	11.2
Other	677	7,059	9.5
Unknown	180	2,362	7.6
Total	12,364	106,808	11.6

### Length of Time Free from CVD Event after Healthy Pregnancies vs. Pregnancies Complicated by PE

Time free from CVD is not equal among the groups (Table III). Overall time to CVD in ADSW is quicker in those who had PE compared to those who did not (HR 1.257), and PE had a significant effect on overall CVD risk. Analysis of time free from CVD by race, age, and service branch by Cox proportional hazard models reveal that both age and service branch have a significant impact on the event-free probability time with a significant difference between the highest and lowest groups. Time to CVD decreases as age increases, with women aged 35+ having the highest overall HR in this analysis. Using Asians as referent, race also has a significant impact with African Americans at highest risk (HR 1.382) followed by Whites (HR 1.322).

**TABLE III.** Time to CVD Calculated by Cox Hazard Model

Referent group	Effect	Hazard ratio	*P*-value
Preeclampsia (referent: No)	PE (Yes vs. No)	1.257	<0.0001
Age group (referent: ≤24yo)	Age group (25–29)	1.158	0.0012
	Age group (30–34)	1.373	<0.0001
	Age group (35+)	1.628	<0.0001
Race (referent: Asian)	Race (White)	1.322	0.0002
	Race (African American)	1.382	<0.0001
	Race (Other)	1.228	0.0336
Service (referent: Navy)	Service (Army)	1.254	<0.0001
	Service (Air Force)	1.393	<0.0001

## DISCUSSION

To the best of our knowledge, this is the first case-control study investigating CVD in the setting of PE among US Military servicewomen. Even with widespread electronic record keeping and a steady but slow increase in reports, the quantity and quality of PE studies have been characterized by paucity of data. Comparison of population-based studies on the incidence of PE with our study suggests that the incidence in the US Military population is higher across all races compared to civilians with the caveat that we are limiting our analysis to inpatient data. A large study utilizing US civilian data from the National Hospital Discharge Survey between 1979 and 2006 that included 791,764 deliveries reported a PE rate (not including eclampsia) of 4.17% for African American women and 2.79% for Caucasian women.^7^ Thus, the PE rate is ∼ 3.5-fold lower for Caucasian civilian women than ADSW and ∼2.4-fold lower for African American civilian women than ADSW identified in our study. Similarly, another US-based study from Washington State of 794,648 study participants (79% Caucasians, 9.1% Hispanic, 3.2% African American, and 8.7% other) reported a combined incidence of any form of PE/eclampsia of 2.58%; that is lower than our study.^8^

Another important observation of this report is the relatively small difference (0.2%) between the PE rates between Caucasian and African American women in our study population. In contrast, the abovementioned study by Breathett et al. observed a higher PE rate in the civilian population African American group: 4.17% for African Americans and 2.79% for Whites.^7^ While the numbers for both races are higher than reported civilian population, the difference between the races is much smaller in our cohort.

Mitigation of racial disparities in servicemembers is not unique to our study. Pope et al. performed a retrospective cross-sectional study of MHS data in 2011–16, limiting their analysis to the direct care system similar to our study.^9^ They found that while 49.4% of White Active-Duty Women participated in cervical cancer screening, so did 50.7% of African American Active Duty Women, which is in stark contrast to the civilian population where African American women are 50% less likely to be screened than White women.^10^ In civilian populations, breast cancer screening, like cervical cancer screening, is less accessed by African American women than White women.^12,13^ A study by Bytnar et al. analyzed the impact of guideline changes for breast cancer screening on participation among women of the US Military between 2010 and 2015. Similar to our study, Bytnar et al compared races and military branches^11^ by analyzing how many women screened/10,000/quarter. They reported for the age group 40–49 years 462 White and 444 African American women and in the age group of 50–64 years 533 White and 551 African American women (all at baseline). All groups developed similarly over time in response to the guideline change and the authors discuss this lack of racial disparity of US Military women as “perhaps the most relevant finding of this study.”

We found only one study with acceptable quality CVD follow-up data that differentiated between different US civilian races, the abovementioned study conducted in Washington State by Kestenbaum et al.^8^ The authors found that African American women are at a significantly higher risk for CVD events after a hypertensive pregnancy (*P* < 0.001) while the risk for Hispanics and Other ethnicities was not significantly different using Whites as a reference. In contrast, in our study, we find White and African American Active-Duty Military women to have similar times of event free probability and both lower than, if not significantly so, than Asian women (see Fig. 1, *P* = 0.00043). Several studies published from around the world do not differentiate between different races. A Norwegian study with 617,589 deliveries, a PE rate of 4.89%, and a median follow-up time of 14.3 years reported that 6.14% of women with PE needed hospitalization for CVD compared with 3.05% for the control population, and 0.4% of the women died from CVD vs. 0.25% for control population. Interestingly, the reported risks for women who had a pregnancy with gestational hypertension presented with the same risks as those with PE which was beyond the scope of our analyses.

Service affiliation may have an impact on CVD event-free probability time. We observed women in the Air Force at the highest risk and women in the Navy at the relatively lowest risk (Fig. 1C, *P* < 0.0001). Pope et al. also found significant differences in the percentage of women undergoing cervical cancer screening with women in the Navy at 84.4% being the most active participants followed by the Army with 45.8%, Air Force 40.9%, and the Marines 11.7%. The breast cancer screening study by Bytnar et al. also differentiated between the services and found as follows (age 40–49, screens/10,000): Navy: 508, Air Force: 495, and Army: 415. Thus, ∼18% fewer Army women/10,000 screens each quarter choose to be screened for breast cancer compared to the Air Force: the opposite finding of cervical cancer screening participation. Gen Robert W. Cone wrote in 2016 regarding “Leading Gender Integration” that “a frequently asked question by women candidates was the name of the battalion and brigade commanders who led these units… The importance of unit leadership is again emphasized in virtually every U.S. Army Training and Doctrine Command (TRADOC) survey conducted on gender integration. Very clearly, women identify leadership as one of the critical factors that support the success of gender integration efforts.”^14^ Given the size of the US Military, there may be units within one service where participation in preventative care is more encouraged than in others, directly by active leadership engagement. It is important to identify which measures are most effective in supporting ADSW to promote preventative health-care measures that maintain a ready force.

### Strengths and Limitations

This case-control study of US Military ADSW is the first to investigate CVD risk after a PE pregnancy representing a large study population that also has equal access to care. One limitation of our CVD event data is the expansive breadth of ICD codes included in this group ranging from chronic hypertension to myocardial infarction. While we could have used more defined subgroups, we decided against this strategy as our main focus here was to examine the full scope of CVD events across different racial backgrounds. Another limitation is the analysis of only inpatient data which makes comparison to civilian data, which include both inpatient and outpatient data, more challenging. Inpatient data were of higher quality and the main goal of the study was to generate dependable, accurate numbers for the US Military. We recognize that the universal access to care resources for pregnant ADSW may increase the incidence of PE diagnosis due to better monitoring. Lastly, we recognize the inherent limitations with retrospective data analyses that limit our findings to associations only rather than causation.

## CONCLUSION

PE prevalence among ADSW appears higher than reported civilian populations, but racial differences between African American and White servicewomen are mitigated. CVD disease risk is significantly impacted by the PE pregnancy, and race, age, and service all contribute individually to the specific risk of the service member. Further study of contributing factors, comorbidities, potential preventative strategies, and appropriate monitoring for these military women is warranted to mitigate CVD incidences that can put both the Service member themselves and overall readiness at risk. Recent congressional mandates, such as extended postpartum maternity leave and 12-month deployment deferments postpartum could potentially decrease stress for ADSW, and future studies may assess the impact of these measures on post-PE long-term disease trajectories for these Military Women.

## Data Availability

None declared.

## INSTITUTIONAL CLEARANCE

Institutional clearance approved.
